# A single *Danio rerio hars* gene encodes both cytoplasmic and mitochondrial histidyl-tRNA synthetases

**DOI:** 10.1371/journal.pone.0185317

**Published:** 2017-09-21

**Authors:** Ashley L. Waldron, Sara Helms Cahan, Christopher S. Franklyn, Alicia M. Ebert

**Affiliations:** 1 Department of Biology, University of Vermont, Burlington, VT, United States of America; 2 Department of Biochemistry, University of Vermont College of Medicine, Burlington, VT, United States of America; University of Melbourne, AUSTRALIA

## Abstract

Histidyl tRNA Synthetase (HARS) is a member of the aminoacyl tRNA synthetase (ARS) family of enzymes. This family of 20 enzymes is responsible for attaching specific amino acids to their cognate tRNA molecules, a critical step in protein synthesis. However, recent work highlighting a growing number of associations between ARS genes and diverse human diseases raises the possibility of new and unexpected functions in this ancient enzyme family. For example, mutations in HARS have been linked to two different neurological disorders, Usher Syndrome Type IIIB and Charcot Marie Tooth peripheral neuropathy. These connections raise the possibility of previously undiscovered roles for HARS in metazoan development, with alterations in these functions leading to complex diseases. In an attempt to establish *Danio rerio* as a model for studying HARS functions in human disease, we characterized the *Danio rerio hars* gene and compared it to that of human *HARS*. Using a combination of bioinformatics, molecular biology, and cellular approaches, we found that while the human genome encodes separate genes for cytoplasmic and mitochondrial HARS protein, the *Danio rerio* genome encodes a single *hars* gene which undergoes alternative splicing to produce the respective cytoplasmic and mitochondrial versions of Hars. Nevertheless, while the *HARS* genes of humans and *Danio* differ significantly at the genomic level, we found that they are still highly conserved at the amino acid level, underscoring the potential utility of *Danio rerio* as a model organism for investigating HARS function and its link to human diseases *in vivo*.

## Introduction

Aminoacyl tRNA synthetases (ARS) comprise a family of enzymes responsible for attaching amino acids to their appropriate tRNA molecules, an early step in protein synthesis [1]. For each amino acid there is a dedicated ARS, each of which catalyzes a highly specific reaction in which an amino acid is ligated to one of its cognate tRNA molecules, thereby ensuring the fidelity of the genetic code [1–4]. Proper protein synthesis depends on the ability of these enzymes to accurately and efficiently carry out this activity, making them vital proteins. Interestingly, it is now clear that many of these enzymes also carry out important activities beyond tRNA charging, such as transcriptional and translational regulation, immune response modulation, and signal transduction [5–8]. These additional functions of aminoacyl tRNA synthetases may help explain the growing number of diseases associated with this family of enzymes [9, 10]. The connection between these ubiquitous, essential proteins and the variety of disorders resulting from their deficiency is unclear and indicates a need for further investigation into the many roles of aminoacyl tRNA synthetases.

Here, we focus on histidyl-tRNA synthetase (HARS), which has been implicated in several human disorders, two of which affect different regions of the nervous system [11–13]. The first is Usher Syndrome Type IIIB, a combined deafness-blindness disorder, which is associated with a recessive missense mutation in *HARS* [11]. The other is Charcot-Marie-Tooth (CMT) peripheral neuropathy, which is associated with several different dominant *HARS* missense mutations [12, 13]. How these mutations result in neurological disorders is not known, however they suggest that HARS might serve specific roles in the development and maintenance of nervous tissues.

The exploration of potential alternative functions of HARS in nervous system development would benefit from the availability of animal models that are well-validated as human disease models and have tractable genetics. The zebrafish, *Danio rerio*, has proven to be an excellent model for investigating mechanisms of development and degeneration, and our goal is to use this model to help elucidate the roles of HARS in these processes [14]. Previously, an unbiased screen of genes in *Danio* required for development identified five different ARS genes [15]. With that precedent in mind, we set out to characterize the genetics of *Danio rerio hars*, relative to human *HARS*. Given that genetic homology between *Danio rerio* and humans is a frequently cited asset of the fish model, we predicted that *Danio rerio* and human HARS would be highly similar. Using bioinformatics and molecular phylogenetics we uncovered important insights into the evolutionary history of *HARS* genes. Additionally, our analysis identified important regulatory differences between human *HARS* and *Danio rerio hars*. These results will be valuable both in understanding the roles of Hars in the zebrafish as well as how to establish the zebrafish as a model for studying HARS-based human disorders.

## Materials and methods

### HARS RNA expression in *Danio rerio*

All procedures were approved by the University of Vermont Institutional Animal Care and Use Committee Protocol Number: 14–053 and the University of Vermont Institutional Biosafety Committee Protocol Number: 14–024. Fertilized AB embryos were raised at 28.5°C and staged as previously described [16]. Total RNA was isolated from manually dechorionated embryos at 48 hours post fertilization using Trizol:chloroform (Invitrogen, California, USA). Using this RNA, cDNA was generated by Superscript II Reverse Transcriptase (Invitrogen, California, USA) as described in the product manual. The following primers were used to amplify HARS (note that these primers were designed to add Sp6 and T7 promoters to the 5’ and 3’ ends of the product respectively):

*hars* Forward: 5’–ATTTAGGTGACACTATAGCAAAAGTGAGAAAGCGAGCA -3’

*hars* Rev: 5’–TAATACGACTCACTATAGGGTCAGGGATCATTGCATCGTA -3’

Following PCR amplification the products were cloned into the pCR-Blunt II-TOPO vector (Invitrogen, California, USA) and sequenced in the University of Vermont Cancer Center Advanced Genome Technologies Core using M13 forward and reverse primers.

### Sequence analysis and phylogenetics

Table 1 shows the NCBI RefSeq ID numbers used for protein subcellular localization prediction and amino acid sequence alignments. Programs used for subcellular localization are described in the results. For the amino acid sequence alignments between human and *Danio rerio* proteins, sequences were aligned with MAFFT using default settings [17]. Domain regions were determined using the Conserved Domain tool from NCBI [18]. The following sequences were used for the alignments of aminoacyl tRNA synthetases other than HARS: AARS NP_001037775.1, NP_001596.2; CARS NP_001112372.1, NP_001014437.1; DARS NP_001071079.1, NP_001340.2; EPRS NP_001275581.1, NP_004437.2; FARSA NP_001038760.2, NP_004452.1; FARSB NP_001007769.1, NP_005678.3; GARS XP_017209451.1, NP_002038.2; IARS NP_956190.2, NP_002152.2; KARS NP_001002386.1, NP_001123561.1; LARS XP_698279.6, NP_064502.9; MARS NP_956370.1, NP_004981.2; NARS XP_696748.4, NP_004530.1; QARS NP_957507.1, NP_005042.1; RARS NP_956342.1, NP_002878.2; SARS NP_001003882.1; NP_001317598.1; TARS NP_001116258.1, NP_001245366.1; VARS NP_001298275.1, NP_006286.1; WARS NP_957066.1, NP_004175.2; YARS NP_958473.1, NP_003671.1.

**Table 1:** RefSeq ID numbers for HARS and Cox8a protein sequences used for subcellular localization and sequence alignments.

Species	Protein	RefSeq Numbers
Danio rerio	Hars-001	NP_001289191.1
Danio rerio	Hars-002	NP_001289185.1
Danio rerio	Cox8a	NP_001289982.1
Homo sapiens	HARS	NP_002100.2
Homo sapiens	HARS2	NP_036340.1

To reconstruct molecular evolution and gene gains and losses, mRNA sequences of *HARS* and *HARS2* genes for two representative species of mammals, birds, fish, invertebrates and an outgroup (yeast) were downloaded from the NCBI RefSeq database (Table 2). The nucleotide sequences for the *HARS* and *HARS2* genes were trimmed to include only the gene coding (CDS) regions and translated. The amino acid sequences were aligned with Muscle using the default settings and then untranslated for phylogenetic analysis [19]. The best model of evolution (GTR + Gamma) was determined using ML model selection in MEGA5 [20]. This model was used to build a ML tree tested for statistical support with 1000 bootstrap replicates.

**Table 2:** RefSeq ID numbers of HARS and HARS2 mRNA sequences used for molecular phylogenetic analysis of the HARS gene family in animals.

Species	Gene	RefSeq Number
Caenorhabditis elegans	hars-1	NM_001028202.4
Danio rerio	*hars*	NM_001302256.1
Drosophila melanogaster	HisRS	NM_001272756.1
Gallus gallus	HARS	NM_001006144.1
Gallus gallus	HARS2	XM_001231931.4
Homo sapiens	*HARS*	NM_002109.5
Homo sapiens	*HARS2*	NM_012208.3
Saccharomyces cerevisiae	HTS1	NM_001184130.1
Taeniopygia guttata	HARS (Predicted)	XM_012575990.1
Taeniopygia guttata	HARS2 (Predicted)	XM_012575522.1
Takifugu rubripes	*hars*	XM_011618127.1
Xenopus tropicalis	hars2	XM_018092588.1
Xenopus tropicalis	hars	XM_018092607.1
Nanorana parkeri	hars (Predicted)	XM_018561908.1
Nanorana parkeri	hars2 (Predicted)	XM_018561914.1

To test whether the reconstructed phylogeny supported multiple independent origins of HARS2, the resulting best tree was tested against a tree in which HARS2 in amphibians, birds and mammals were constrained to be monophyletic with a Shimodaira-Hasegawa (SH) test in RAxML [21].

### HARS expression constructs

We obtained the single *Danio rerio* Genome Collection full-length *hars* ORF clone, contained in the mammalian expression vector pExpress-1 (Dharmacon, Colorado, USA; Clone ID 7052125). Comparison of this ORF to sequences available from NCBI suggested that there was an A missing near the 3’ end of the sequence (NM_001302256.1, NM_001302262.1). We used the QuikChange II XL Site-Directed Mutagenesis Kit (Agilent Technologies, California, USA) to add an A at this site as per the manufacturer’s protocol and the following primers:

Forward: 5’—GTG GGG ATG TTT GAC CCC AAA GGC AGG AAA GT—3’

Reverse: 5’—ACT TTC CTG CCT TTG GGG TCA AAC ATC CCC AC—3’

The resulting sequence was predicted to encode the amino acid sequence identical to HARS variant 1 (NP_001289185.1). In order to make a construct for HARS variant 2 (NP_001289191.1), a fragment consisting of a portion of the pExpress-1 vector from the SacII restriction site to the insert, followed by the 5’ end of variant 2 up to the ClaI restriction site in *hars* was synthesized (BioBasic). Using standard cloning procedures, we replaced the analogous region in the plasmid with this synthesized fragment and sequenced by the University of Vermont Advanced Genome Technologies Core to confirm that the new construct matched that of *hars* variant 2. Having made constructs for both *hars* variants, we sub-cloned the open reading frame of each into pCMV6-Entry-Myc-DDK vector (Origene, Maryland, USA) such that that they were in frame with the sequence for the DDK (or FLAG) tag.

### Cell culture and transfection

COS7 (gift of John Blenis, Weil Cornell Medical School), were maintained in Dulbecco’s Modified Eagle’s Media (Invitrogen, California, USA) supplemented with 5% Fetal Bovine Serum (Hyclone, Logan, UT, USA), 5% Cosmic Calf Serum (Hyclone, Logan, UT, USA), 50 U/mL penicillin, and 50 μg/mL streptomycin, at 37°C and 5% CO_2_. Cells were co-transfected with 0.25 μg of the pDsRed2-mito plasmid (gifted to us by Dr. Alan Howe, University of Vermont) as well as 1.25 μg of either *hars-001*-FLAG or *hars-002*-FLAG plasmids (construction described above) using polyethylenimine. Plasmids were mixed with 4.5 μL of polyethylenimine (1 mg/mL) and Optimem (Invitrogen, California, USA) to a total volume of 150 μL and incubated at room temperature for 20 min, before 130 μL were added dropwise to cells grown to approximately 70% confluency on glass coverslips in six well plates. After cells had incubated with the transfection reagents for 24 hours they were processed for immunocytochemistry.

### Immunocytochemistry

Transfected cells were fixed in 3.7% Paraformaldehyde in PBS (Electron Microscopy Sciences, Pennsylvania, USA) for 10 minutes, permeabilized in 0.5% Triton X-100 in PBS for ten min and then blocked in 1.5% BSA. The cells were incubated for one hour with anti-FLAG M2 monoclonal antibodies (Sigma, Missouri, USA; Cat # F1804) at 1:5000 in 1.5% BSA. After washing away primary antibody, AlexaFluor 488 conjugated anti-mouse antibodies (Cell Signaling Technology, Massachusetts, USA; Cat # 4408S) were added at 1:10,000 in 2% BSA and the cells were incubated for 45 minutes at 4°C. Cells were washed with PBS and mounted on coverslips with Vectashield with DAPI (Vector Labs, USA).

### Imaging

Expression of HARS-FLAG and mito-DsRed was imaged on a Nikon Eclipse Ti confocal microscope using a 40x objective. Single plane images were captured using Nikon Elements software. Images were processed with Adobe Photoshop to merge channels and adjust overall brightness and contrast.

## Results

### A single *Danio rerio hars* gene encodes two distinct transcripts

In order to meet the universal demand for efficient protein synthesis, eukaryotic organisms require a complete set of aminoacyl tRNA synthetases for each compartment where translation occurs (i.e. the cytoplasm and the mitochondria). This is accomplished using different strategies, depending on the type of ARS and on the organism in question. The human genome provides a representative example. For most ARS families, the genome encodes separate genes for each of these two compartments. In the human genome, this is true of 17/20 of the canonical ARS families (Table 3) the three exceptions being glycyl-tRNA synthetase (GARS), lysyl-tRNA synthetase (KARS) and glutaminyl-tRNA synthetases (QARS). For these exceptions, cells use alternative methods to fulfill translational demands in both the cytoplasm and mitochondria [22–24].

**Table 3:** The number of aminoacyl tRNA synthetase genes in human and *Danio rerio*.

Aminoacyl-tRNA Synthetase	Number of Human Genes	Number of *Danio rerio* Genes	Aminoacyl-tRNA Synthetase	Number of Human Genes	Number of *Danio rerio* Genes
**HARS**	**2**	**1**	SARS	2	2
GARS	1	1	TARS	3	3
AARS	2	2	CARS	2	2
VARS	2	2	YARS	2	2
LARS	2	2	NARS	2	2
IARS	2	2	QARS	1	1
MARS	2	2	DARS	2	2
WARS	2	2	EARS	2	2
FARS	3	3	KARS	1	1
PARS	2	2	RARS	2	2

Shown is a list of the twenty aminoacyl tRNA synthetases (ARS) and the number of associated genes in humans and *Danio rerio*.

Among the tRNA synthetase families with separate cytoplasmic and mitochondrial orthologs in the human genome is the histidine family, where *HARS* encodes the cytoplasmic version and *HARS2* encodes the mitochondrial enzyme. Analysis of the *Danio rerio* genome indicates a high degree of conservation with respect to ARS gene organization between it and the human genome. The only exception is that in the *Danio rerio* genome only a single gene for *hars* is annotated (Table 3). The absence of a second *hars* gene suggests that either there is another unannotated *hars* gene in the *Danio rerio* genome, or that alternative splicing is employed to generate both enzymes.

We used the NCBI BLAST tool to search for genes in the *Danio rerio* genome similar to mitochondrial *hars* genes from species with separate mitochondrial and cytoplasmic genes. The search revealed just two genes, the previously annotated *hars* and the eukaryotic initiation factor 2 kinase 4 (*eIFAK4*), which is the *Danio rerio* ortholog of the yeast GCN2 kinase. All members of the GCN2 family bind uncharged tRNA during their function as nutritional sensors, and thus possess regions homologous to the catalytic domain of HARS [25]. However, such homologs lack aminoacylation function, and are therefore unable to serve as mitochondrial histidyl-tRNA synthetases. Thus, these search results strongly suggest that *Danio rerio* encodes only a single functional *hars* gene.

**Fig 1:**
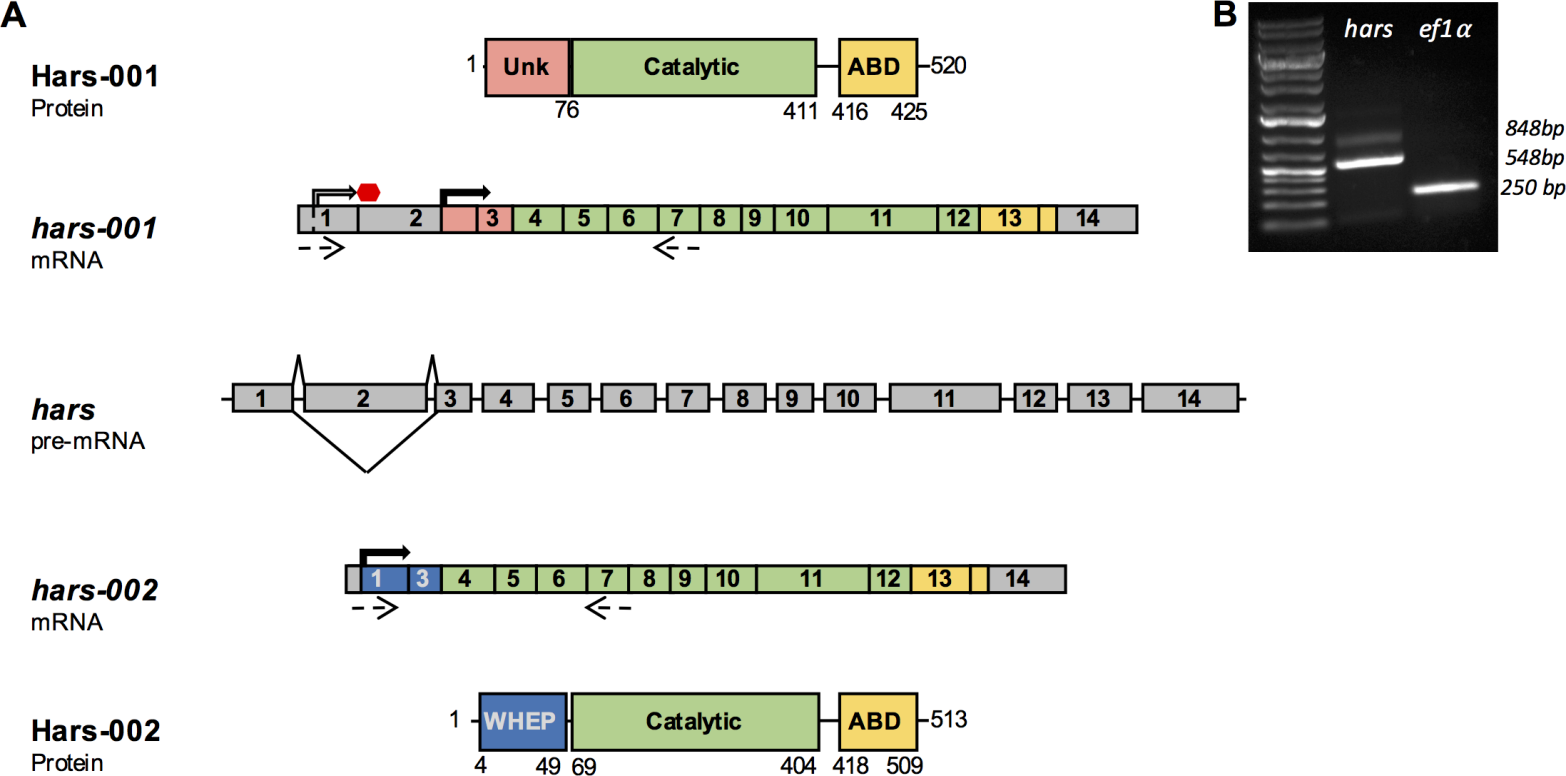
*Danio rerio hars* generates two transcripts that are predicted to code for distinct proteins. (A) Diagram showing the alternative splicing of *hars* pre-mRNA into the two transcript variants and the domain structures of the resulting protein products. Solid arrows indicate translation start sites. Dashed arrows indicate primer binding sites used for B. Unk, unknown; ABD, Anticodon Binding Domain. (B) As shown in lane 2, PCR for *hars* from *D rerio* cDNA using primers noted by the dashed arrows in A generates two products that differ in size by the length of exon 2 (300 bp). Lane 3 is a PCR product for *ef1α*, which was used as a positive control.

There are two mRNA variants listed in the NCBI database for *Danio rerio hars*, consisting of a transcript of 2091 nucleotides (nt) and a second of 1782 nt. Notably, the two transcripts differ by the inclusion or exclusion of a 309 nt exon near the 5’ end of the message; aside from this difference, the sequences are identical. This exon is number 2 in transcript 1, and is absent from transcript 2 (Fig 1A). Using primers complementary to regions shared by both variants we performed RT-PCR on RNA isolated from *Danio rerio* embryos and were able to amplify two products that corresponded to each variant (Fig 1B). We subsequently cloned both amplicons and sequence verified that they corresponded to the predicted splice products.

The inclusion of exon 2 is predicted to alter the protein product of transcript 1 relative to transcript 2. There are two possible translation start sites in exons 1 and 2 of transcript 1 (solid arrows in Fig 1A). However, the first start site in exon 1 is quickly followed by a stop codon in exon 2 (Grey arrow in Fig 1A). The second translation start site in exon 2 is predicted to generate a 520 amino acid protein, which contains both the HARS catalytic and anticodon binding domains (Fig 1A). Because transcript 2 does not contain exon 2, translation would begin at the first start site in exon 1 and produce a 513 amino acid protein. Both start sites are in the same reading frame, meaning that the resulting proteins would be identical except at their N-termini (Fig 1A). The N-terminus of variant 1 is uncharacterized, but variant 2 is predicted to contain a WHEP domain at its N-terminus [18]. This domain is also found at the N-terminus of human HARS (cytoplasmic), but not the N-terminus of human HARS2 (mitochondrial).

### *Danio rerio hars* protein products are differentially localized within the cell

Subcellular targeting sequences are frequently found at the N-termini of proteins, so the fact that these two protein variants differ only at their N-termini suggested to us that they might be differentially localized within the cell. We first addressed the question of whether the two protein products might be differentially localized by using several publically available sub-cellular localization prediction tools. The methods and output of each of these tools varies, but each could assess whether a protein was likely to be targeted to the mitochondria or not. We tested the ability of each of these tools to accurately predict the localization of *Danio rerio* proteins using *Danio rerio* cytochrome c (Cox8a), a known mitochondrial protein as a positive test. All four tools correctly predicted mitochondrial localization for this sequence (Table 4). With respect to Hars, each of the four tools predicted that variant 1 would localize to the mitochondria and variant 2 to a location other than the mitochondria, i.e. the cytoplasm (Table 4). One predictor, MitoFates, identifies specific motifs within the protein sequence that are common among mitochondrial proteins [28]. Using this tool, *Danio rerio* Hars-001 was found to contain four components that strongly suggest mitochondrial targeting and are similar to other proteins destined for the mitochondrial matrix [31].

**Table 4:** Predicted localization of *Danio rerio* genes by four subcellular localization predictor programs.

	YLoc [26, 27]	MitoFates [28]	TargetP [29]	BaCelLo [30]
Hars-001	Mitochondria	Mitochondria	Mitochondria	Mitochondria
Hars-002	Cytoplasm	Not Mitochondria	Other	Cytoplasm
Cox8a	Mitochondria	Mitochondria	Mitochondria	Mitochondria

The table shows the subcellular locations of the two Hars variants and cytochrome c (Cox8a) predicted by the four prediction tools listed in the top row.

**Fig 2:**
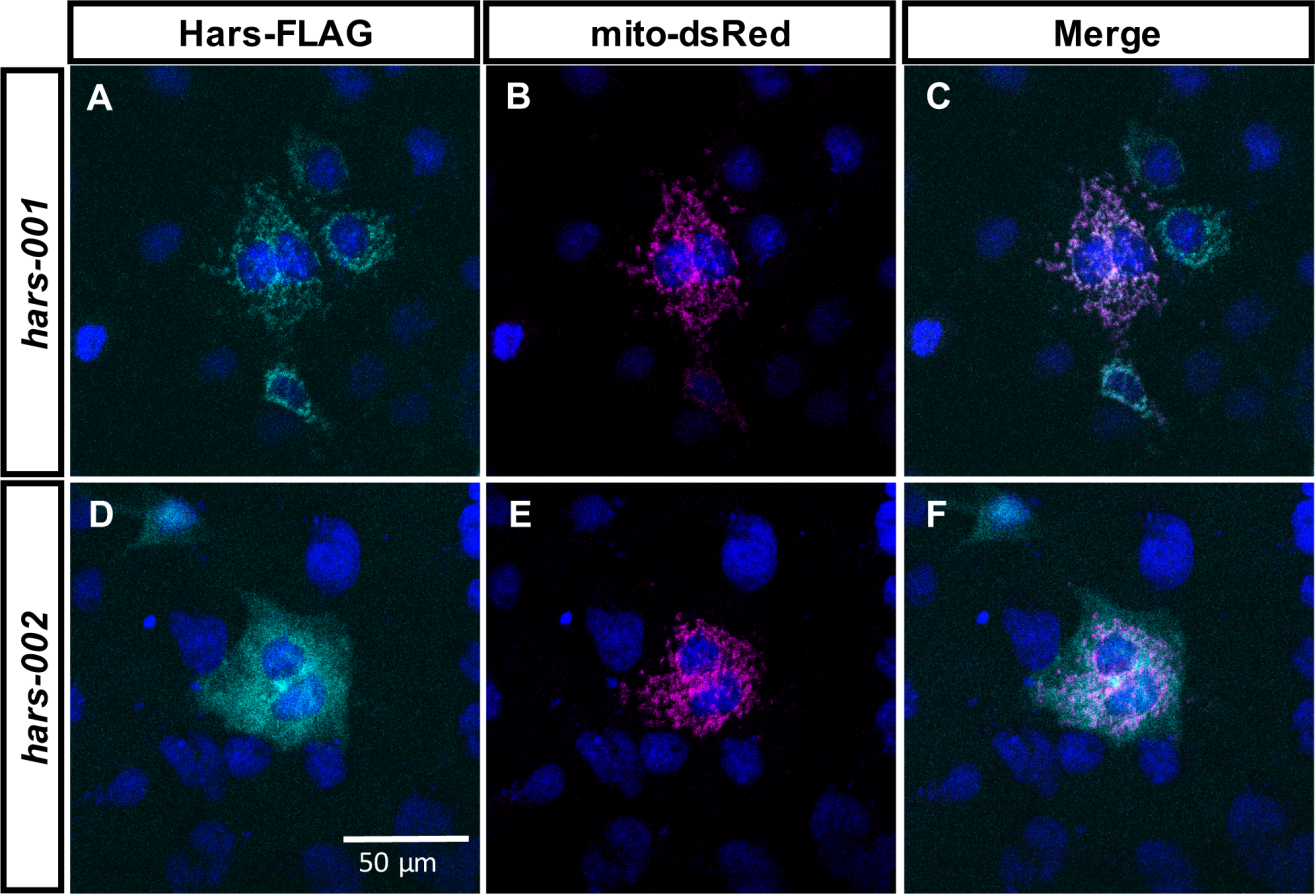
Hars-001 and Hars-002 are differentially localized within cells. (A, D) FLAG immunohistochemistry of COS7 cells transfected with C-terminally FLAG-tagged Hars variants. (B, E) Cells were co-transfected with a mitochondrial-targeted red fluorescent protein (mito-dsRed) to identify mitochondria. (C, F) Merged images show Hars-001-FLAG co-localizes with the mitochondria marker, while Hars-002-FLAG appears more cytoplasmic. Scale bar in F is 50 μm and is the same for all panels.

In order to directly test these predictions experimentally, we generated FLAG-tagged constructs based on the cDNA sequence for each transcript and transfected them into COS7 cells along with a construct for a mitochondrial-targeted red fluorescent protein that has been used previously to mark mitochondria [32]. We performed immunocytochemistry for the FLAG epitope to visualize HARS localization within the cell relative to the mitochondrial marker and found variant 1 co-localized with the mitochondrial marker, while variant 2 spread throughout the cytoplasm (Fig 2). These localization patterns indicate the single *Danio rerio hars* gene does in fact generate both a mitochondrial and cytoplasmic protein through alternative splicing.

### The *HARS* gene was duplicated in vertebrates after the divergence of teleosts

**Fig 3:**
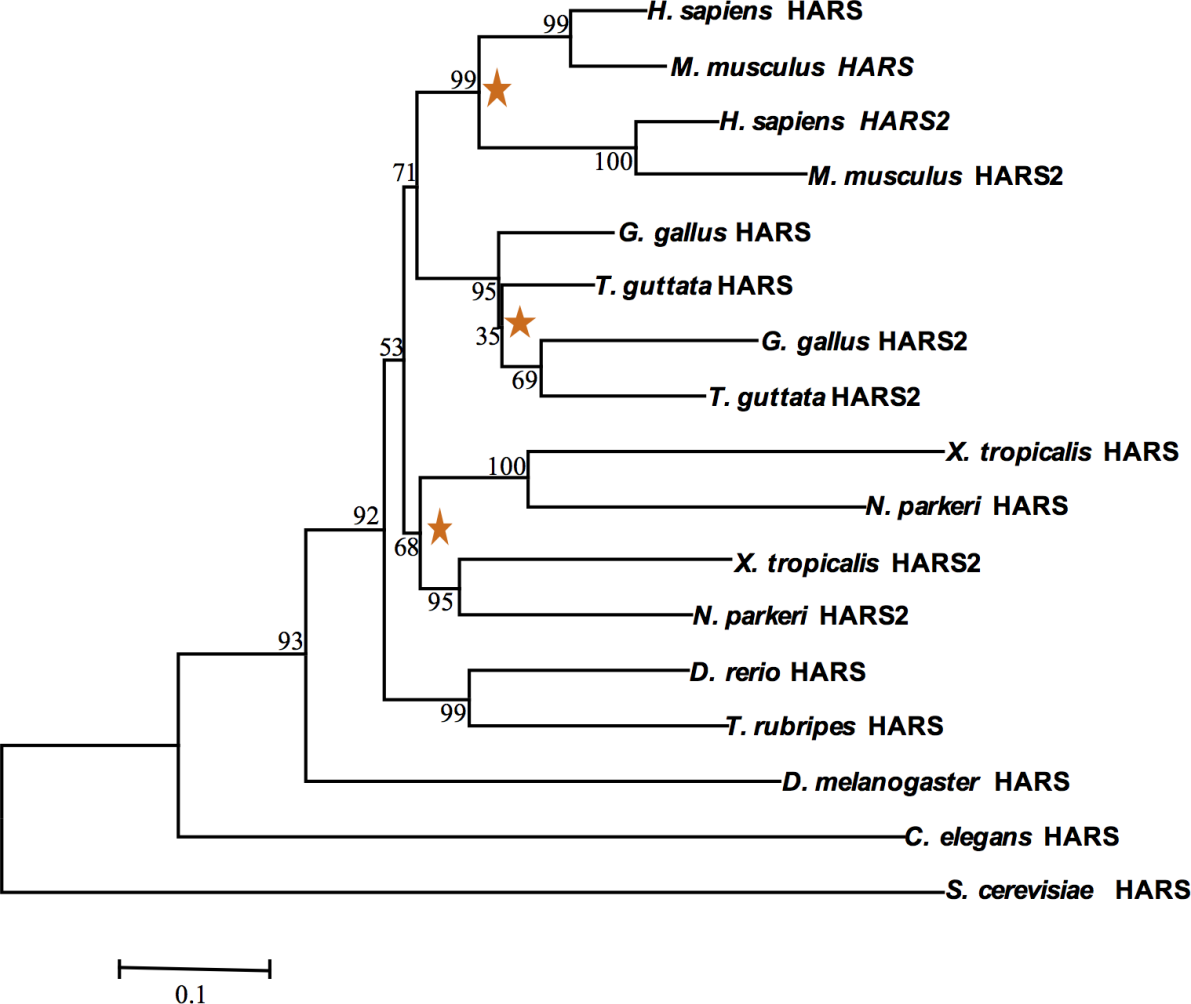
Phylogenetic tree for *HARS* suggests multiple duplication events in *HARS* evolutionary history. A maximum likelihood tree constructed using the mRNA sequences of *HARS* and *HARS2* (see Materials for RefSeq numbers) shows the relatedness between *HARS* transcripts among representative species. Stars represent putative duplication events; numbers at each node are percent bootstrap support out of 1000 replicates.

**Fig 4:**
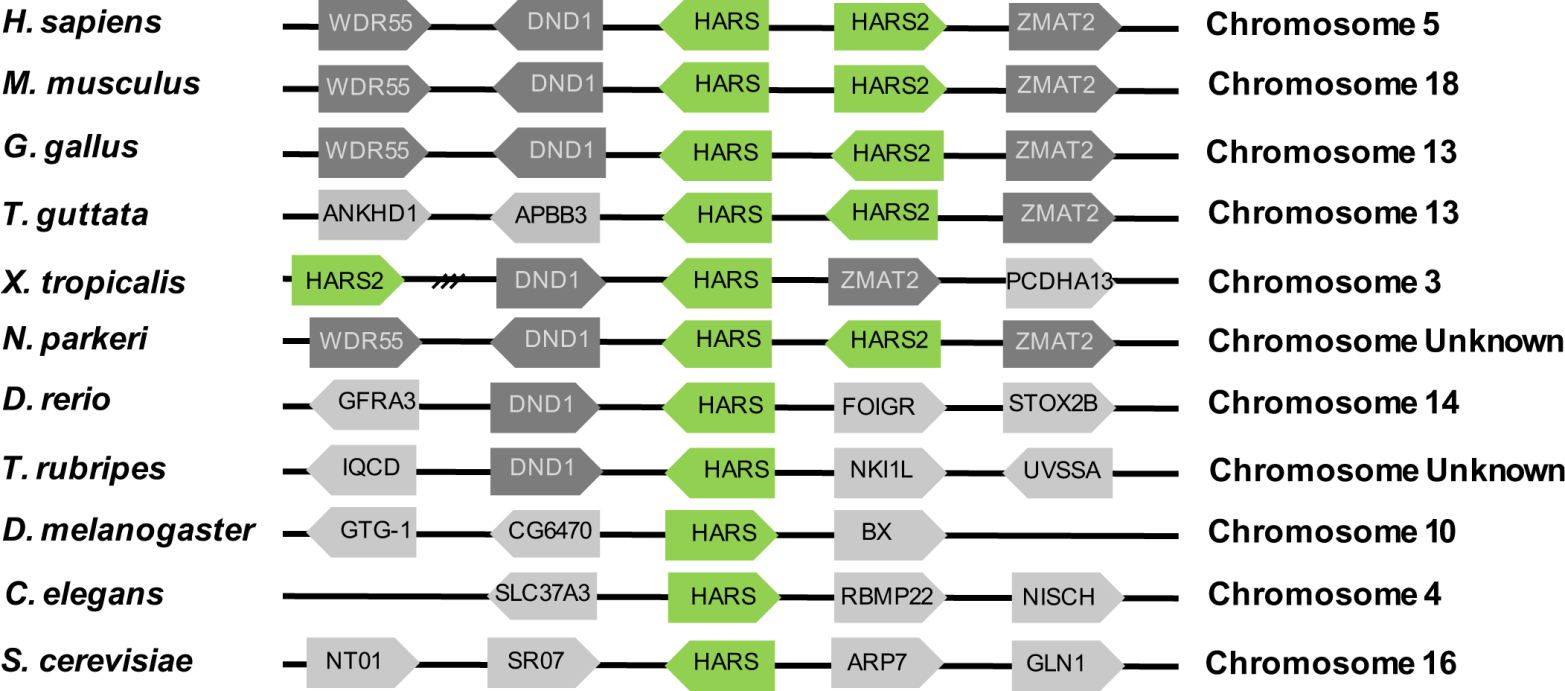
Synteny of *HARS* genes suggests that *HARS* has been duplicated multiple times in the animal kingdom. The chromosomal positioning of *HARS* genes reveal clade-specific patterns, with the exception of amphibians. For example, mammalian *HARS* and *HARS2* are arranged back-to-back, while bird *HARS* genes are side-by-side but with the same orientation.

To reconstruct the evolutionary history of *HARS* gene duplication, we analyzed patterns of *HARS* and *HARS2* mRNA sequence divergence across representative species of the major clades of vertebrates, along with invertebrate and yeast outgroups. Monophyly of the *HARS* gene family in vertebrates was strongly supported (Fig 3). The existence of a single *HARS* gene is shared among *D*. *rerio*, *T*. *rubripes*, *D*. *melanogaster*, *C*. *elegans*, *and S*. *cerevisiae*, suggesting that this is the ancestral trait among this group of eukaryotes. It has been shown that *S*. *cerevisiae* use alternative transcripts to produce both a mitochondrial and cytoplasmic HARS protein, and has been predicted for the other species [33, 34] The presence of two *HARS* genes appears to be unique to higher vertebrates, starting at the level of amphibians (Fig 3). Interestingly, however, the cytoplasmic and mitochondrial *HARS* genes clustered by clade rather than by function, and the hypothesis of monophyly of *HARS2* was rejected (SH test, P < 0.01), suggesting multiple independent origins. Separate duplication events in amphibians, birds and mammals are also supported by clade-level variation in chromosomal arrangement of these genes (Fig 4). These patterns intuitively fit with those seen in our phylogenetic analysis.

### *Danio rerio* and human HARS amino acid sequences demonstrate conservation

**Fig 5:**
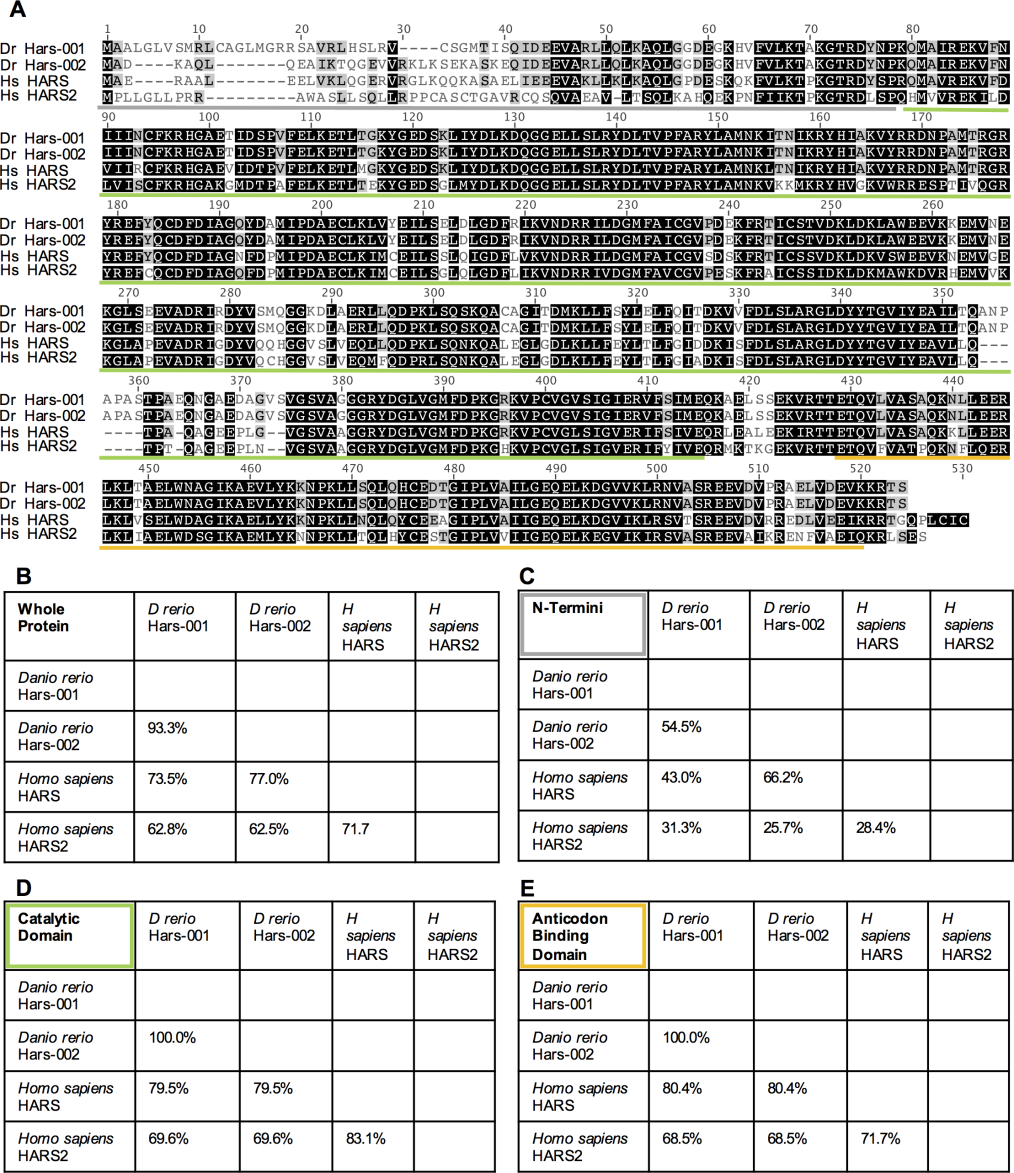
Protein alignment between *Danio rerio* and *Homo sapiens hars*. Whole protein alignment of *Homo sapiens* HARS and HARS2 with *Danio rerio* HARS-001 and HARS-002 shows the high degree of identity among the proteins. Colored bars indicate the various domains as seen in Fig 1. (B-E) Percent identity matrices for the whole protein alignment seen in A as well as for each domain separately (domains denoted by colored bars in A).

Amino acid sequence alignments of the *Danio rerio* and human HARS proteins show the two *Danio rerio* Hars splice variants share 73.3% (Hars-001) and 77% (Hars-002) amino acid identity with the human cytoplasmic HARS and about 62% with human mitochondrial HARS2 across the whole protein (Fig 5A and 5B). We also aligned each domain separately and, with exception of the N-termini, found similar levels of identity (Fig 5C, 5D and 5E). As discussed previously, the N-termini specify subcellular localization and therefore were expected to be less similar to one another. The overall similarity between the *Danio rerio* Hars proteins and human HARS and HARS2 suggests that despite the genomic differences the enzymes are conserved. The identity between the cytoplasmic HARS proteins of *Danio* and humans (77%) is similar to the percent identity observed between other cytoplasmic ARS of these two species (Table 5), and several of these enzymes have already been studied using *Danio rerio* [35–39].

**Table 5:** Percent identity between the amino acid sequences of human and *Danio rerio* cytoplasmic aminoacyl tRNA synthetase homologues.

Aminoacyl-tRNA Synthetase	Percent Identity between Human and *Danio rerio*	Aminoacyl-tRNA Synthetase	Percent Identity between Human and *Danio rerio*
**HARS**	**77%**	SARS*	78%
GARS*	81.6%	TARS*	84.5%
AARS	81.8%	CARS	73.6%
VARS	65.2%	YARS*	81.9
LARS	79.7%	NARS	81.8%
IARS	74.1%	QARS*	41.8%
MARS	63.1%	DARS	78.2%
WARS	74.3%	EPRS	66.4%
FARSA	77.9%	KARS	75.8%
FARSB	75.8%	RARS	76.6%

HARS has a similar if not higher percent identity than other synthetases, several of which have already been studied using *Danio rerio* (asterisks).

## Discussion

The goal of this study was to gain a better understanding of the *Danio rerio hars* gene in order to use this species as a model for studying functions of HARS. Over the course of their evolution, aminoacyl-tRNA synthetases have accumulated a number of auxiliary domains, many of which are connected to their various non-canonical functions [40]. It was entirely possible that in the time since the *Danio rerio* and human branches diverged HARS could have accumulated major differences, such as extra domains. What we found was that these two species’ HARS proteins share a high degree of identity at the amino acid level. However, functional and structural analyses would be necessary to confirm that *Danio* and Human HARS proteins share similar functions.

We also show that *Danio rerio* and humans code for HARS proteins by different genetic mechanisms. We confirmed that *Danio* rerio utilize a single *hars* gene to produce both a cytoplasmic and mitochondrial enzyme, while humans have separate genes. Interestingly, we found that *Danio rerio* is unique among the queried vertebrates in having a single *HARS* gene and this appears to be an ancestral condition as this pattern is also observed in invertebrates and yeast. We had expected to find that among the vertebrates that do have two *HARS* genes there would be evidence of a single duplication even that occurred after the teleosts arose (represented by *Danio* and *Takifugu* in Fig 3). What we and others have found is that it appears that *HARS* has been duplicated multiple times in different eukaryotic groups [41]. In most cases, one of the gene products was predicted to be cytoplasmic while the other was predicted to be mitochondrial when analyzed by the subcellular localization tools used for predicting the localization of the two *Danio rerio* variants. This leads to the question of why would separate genes for cytoplasmic and mitochondrially targeted HARS proteins arise multiple times, when organisms clearly had the ability to make both from a single gene? One possibility is that it allows for more precise transcriptional regulation of the two proteins. There is little known about regulation of *HARS* genes. However, by looking at expression data available from the mouse Gene Expression Database it appears that *Hars* and *Hars2* are differentially expressed [42]. The functional relevance of these expression differences is yet to be determined.

*HARS* duplication could also allow functional differences between the mitochondrial and cytoplasmic proteins to arise after duplication. Lee, et al. found that in the case of humans, HARS and HARS2 have adopted divergent tRNA recognition properties, such that HARS is highly specialized to recognize only the cytoplasmic tRNA^His^ while HARS2 only recognizes the mitochondrial tRNA^His^ [41]. In contrast, they show that HARS proteins from species with a single gene, such as *S*. *cerevisiae*, *D*. *melanogaster*, *and C*. *elegans*, are able to aminoacylate both cytoplasmic and mitochondrial tRNA^His^ [41]. *Danio* Hars proteins may exhibit similar tRNA recognition and aminoacylation to these other species with bifunctional HARS, however functional tests are needed to confirm this prediction.

As we have mentioned, we are ultimately aiming to use *Danio rerio* for functional studies on Hars in hopes that it helps us better understand HARS-related disorders. Because a single gene produces both proteins in *Danio rerio*, creating mutations in *hars* that are homologous to human mutations would effectively cause mutations in both the cytoplasmic and mitochondrial proteins, which could confound the results of these genetic manipulations. We believe that despite this caveat, zebrafish will still make a valuable model, as they are vertebrates amenable to genetic manipulations, develop rapidly and externally, there are a wealth of transgenic lines that allow visualization of our tissues of interest [14]. Others have successfully used *C*. *elegans*, which also only have one *hars* gene, to assess the effect of *HARS* CMT mutations on neuronal development [12,13]. By performing future studies in *Danio rerio* we could provide an even more relevant model for studying neurodevelopmental and degenerative mechanisms.

HARS, as with other aminoacyl tRNA synthetases, is undoubtedly a critical enzyme in all cells and branches of life. Like other synthetases, HARS has likely acquired novel functions throughout evolution as evidenced by its intriguing ties to human disorders and the presence of a metazoan-specific domain (the N-terminal WHEP domain) [11, 12, 13, 43]. This research provides critical background necessary to establish *Danio rerio* as a model for studying HARS function *in vivo*, which will inevitably provide a further understanding of the diverse roles that aminoacyl tRNA synthetases play in normal and disease states.
