# Correction: A single *Danio rerio hars* gene encodes both cytoplasmic and mitochondrial histidyl-tRNA synthetases

**DOI:** 10.1371/journal.pone.0190757

**Published:** 2018-01-02

**Authors:** Ashley L. Waldron, Sara Helms Cahan, Christopher S. Francklyn, Alicia M. Ebert

The third author’s name is spelled incorrectly. The correct name is: Christopher S Francklyn. The correct citation is: Waldron AL, Cahan SH, Francklyn CS, Ebert AM (2017) A single *Danio rerio hars* gene encodes both cytoplasmic and mitochondrial histidyl-tRNA synthetases. PLoS ONE12(9): e0185317. https://doi.org/10.1371/journal.pone.0185317
